# Talocalcaneal coalition combined with flatfoot in children: diagnosis and treatment: a review

**DOI:** 10.1186/s13018-014-0129-9

**Published:** 2014-12-14

**Authors:** Binghua Zhou, Kanglai Tang, Mark Hardy

**Affiliations:** Department of Orthopedic Surgery, Third Military Medical University Affiliated Southwest Hospital, Gaotanyan Str. 30, Chongqing, 400038 People’s Republic of China; Department of Foot & Ankle Surgery, HealthSpan Physicians Group, 10 Severance Circle, Cleveland Heights, OH 44118 USA

**Keywords:** Coalition, Flatfoot, Children, Diagnosis, Treatment

## Abstract

Talocalcaneal coalition often leads to a flatfoot deformity in children. Previous reports have uncovered many aspects of tarsal coalition and flatfoot respectively, including the etiology, clinical presentation, and diagnostic imaging, as well as treatment. However, the optimum surgical procedure for talocalcaneal coalition combined with flatfoot has not been definitively determined. The nonconformity of treatment options is due to our incomplete knowledge of biomechanics, diagnosis, and indication of treatment for talocalcaneal coalition with flatfoot. The objectives of this review are to provide an overview of the current knowledge about etiology, biomechanics, classification, diagnosis, and treatment options for talocalcaneal coalitions with flatfoot and highlight its therapies in children.

## Introduction

Tarsal coalition is an abnormal connection between the tarsal bones, which is thought to result from a congenital failure of differentiation in the developing fetal foot. The incidence of tarsal coalition has been reported from less than 1% to as high as 13% [1–3]. Patients frequently have more than one coalition in the same foot and 50% of patients are bilateral. The most common coalitions involve the talocalcaneal and calcaneonavicular bone [1]. In some series, talocalcaneal coalitions account for up to 48% of all tarsal coalition cases [4]. This does not appear to be a sex predilection of tarsal coalitions [5].

The calcaneus is held in a valgus position as ossification progresses with a talocalcaneal coalition, which leads to a rigid flatfoot deformity. Subtalar motion is markedly restricted in the talocalcaneal coalitions but is moderately limited in calcaneonavicular and talonavicular coalitions. Bony coalition can be detected on routine radiographs. However, fibrous or cartilaginous coalitions may be difficult to visualize with standard radiograph. Computed tomography (CT) and magnetic resonance (MR) imaging are invaluable for assessment of tarsal coalitions. Resection of coalition is generally accepted for an intractably painful talocalcaneal tarsal coalition. Despite the possibility that symptoms may be relieved with coalition resection, normal foot alignment and muscular imbalances cannot be restored with resection alone [6]. The treatment for talocalcaneal coalitions with flatfoot in children continues to be a topic of debate. In this review, we present the existing knowledge on the etiology, classification, diagnosis, and treatment of the talocalcaneal coalitions with flatfoot and debate whether there is an optimal surgical procedure.

## Clinical symptoms

Talocalcaneal coalitions with flatfoot generally start as a painless decrease in the range of motion of a joint and will often progress to a symptomatic rigid deformity [1]. Patients with talocalcaneal coalition and flexible flatfoot deformity are often asymptomatic with a rectus foot type. Talocalcaneal coalition becomes symptomatic during childhood as the coalition ossifies and restricts subtalar motion [7]. Pain presents in the sinus tarsi when the middle facet of talocalcaneal joint has a solid osseous bridge. Once the posterior facet of talocalcaneal joint demonstrates degenerative changes; the pain is often located medially, inferior to the medial malleolus, at the site of coalition [8]. Talocalcaneal coalitions with a rigid flatfoot deformity will often show symptoms between 12–16 years of age [9,10]. However, some children between the ages of 6–10 years were found to have completely ossified talocalcaneal coalitions [9].

Talocalcaneal coalition is the most common cause of peroneal spastic flatfoot. Peroneal spastic flatfoot is a syndrome typically characterized by limited tarsal joint motion, a clonus response of the evertors, and a pes planus deformity [11]. Muscle spasm in severe patients may also involve the extensor digitorum longus and peroneus tertius muscles; adaptive shortening of peroneal tendons can lead to reflex spasms [12]. Clinical symptoms of the tarsal coalition frequently follow a sequence of sprains or other minor injuries [1,2]. Because the absence of inflammatory cells has been reported in histopathological studies, it is believed that pain is secondary to mechanical stress arising from the periosteum surrounding the ossifying coalition [13]. Collectively, peroneal spasm is the most common clinical symptom in patients with talocalcaneal coalition and associated flatfoot. Most patients have a consistent pain located at the sinus tarsi and over the sustentaculum tali. However, the location of pain varies and pain can be diffuse throughout the hindfoot complex.

## Etiology

Proposed theories related to the etiology of tarsal coalitions can be described as being either congenital or acquired [1]. The most widely accepted of congenital coalitions is the LeBouq’s theory that autosomal dominant inheritance led to the failure of differentiation of embryonic mesenchymal tissue [1,14]. A previous report showed that Asian people have a higher rate of tarsal coalitions. The pattern of foot coalitions in Native American samples can be traced back to the early appearance and widespread geographic distribution populations migrating from Asia [15]. Recently, Pro250Arg point mutation in fibroblast growth factor receptor 3 gene was thought to result in a tarsal coalition [16]. A much higher percentage of coalitions than anticipated in either the general population or the injured athletic population reinforced that there may be an underlying anatomic predisposition [3]. Talocalcaneal coalitions also can be acquired by degenerative joint disease, inflammatory arthritis, infection, fibular hemimelia, and clubfoot deformities [1].

## Biomechanics

During a normal gait cycle, the subtalar joint experiences rotatory and gliding motion of the talus against the calcaneus. Following heel strike, the talocalcaneal joint assumes relative external rotation and valgus with maximum calcaneal eversion during the first half of the stance. During this stance segment, the calcaneus also dorsiflexes and the Chopart articulation abducts. Then, gradual internal rotation and varus takes place during the remaining stance and swing phase [17]. The subtalar joint functions as a torque convertor, which transmits forces from the tibia to the foot. Mobility of the transverse tarsal joint is determined by the position of the subtalar joint, when the hindfoot is everted into a valgus position, and the transverse tarsal joint is unlocked. Talocalcaneal coalitions restrict mobility of the subtalar joint severely, which lead to increased frontal plane range of motion due to compensatory adaptation or lateral ligamentous laxity [18]. In addition, with the presence of calcaneal valgus, the fibula may come into contact with the lateral calcaneal wall during weight bearing and develop a pseudoarticulation [19].

The importance of achieving normal subtalar kinematics during stance cannot be overemphasized. Normal subtalar kinematics attenuates ground reaction impact forces and limits subtalar eversion, which increases the magnitude of the impact loading experienced during locomotion. Tarsal coalition feet displayed reduced peak pressure and loading at the region of the fifth metatarsal head [6]. Electromyography revealed consistent abnormal activity in the peroneal, gastrocnemius, and soleus muscles on both the operated foot and the contralateral side, including either prolonged monophasic activity or biphasic activity [6].

Many of the operative procedures described for tarsal coalitions may improve gait but they do not fully restore a normal physiologic gait. Therefore, foot function assessment preoperatively and postoperatively is critical to determine the efficacy of various surgical procedures for patients with tarsal coalition. Feet with talocalcaneal coalition had significantly higher medial midfoot pressures during walking and running preoperatively, compared to the asymptomatic extremity. However, medial midfoot pressures showed no significant difference during walking but significantly higher during running after the resection of coalition [20]. The feet continued to be subjected to increased loading and torque in their subtalar and adjacent articulations after resection of coalition [17]. In conclusion, isolated resection of a tarsal coalition fails to restore normal subtalar joint kinematics.

## Classification

Classification systems have been proposed for coalitions based on their anatomic location. For talocalcaneal coalition, most coalitions occur in the middle facet; however, posterior and anterior facet coalitions have also been reported [21]. Coalitions can be further classified into fibrous, cartilaginous, and osseous as to the type of tissue making up the coalitions [22]. Bourdet identified four patterns of flatfoot [23]. These classifications are primarily descriptive for coalition and flatfoot respectively, which afford little information for coalition with flatfeet. Downey introduced and added three important parameters into the articular classification system for tarsal coalition, including: the patient age, articular involvement, and the extent of the secondary arthritic changes [24].

- Type IA: extra-articular coalition without secondary arthritis;
- Type IB: extra-articular coalition with secondary arthritis;
- Type IIA: extra-articular coalition without secondary arthritis;
- Type IIB: extra-articular coalition with secondary arthritis.

This classification system combined the characters of both coalition and flatfoot well. It can be used as a framework to discuss recommended surgical procedures and their clinical results.

## Diagnosis

Congenital talocalcaneal coalition is often overlooked in children who first present with foot and ankle pain [8]. Additionally, the differential diagnosis of talocalcaneal coalition is easy to misinterpret in skeletal samplings [25]. Therefore, careful physical examination and radiograph evaluation are required to correctly diagnose the condition.

### Physical examination

A thorough physical examination determines if the causes of the symptoms are related solely to the coalition, secondary arthritic changes, or flatfoot deformity. Tarsal coalitions with a rigid flatfoot are characterized by peroneal muscle spasms and a painful, rigid valgus deformity [12]. The physical examination should focus on these symptoms.

On clinical examination, patients generally have mild to deep pain within the subtalar joint with limitation of passive range of motion of this joint. During open kinetic chain examination, these patients have a valgus position of the hindfoot, as well as an equinus position at the level of the ankle, forefoot pronation, and loss of medial longitudinal arch height [8]. If the coalition is unilateral, this position is easily observed relative to the contralateral limb.

A toe-raise test can be used to assess the mobility of the hindfoot. The patient is asked to rise on the toes of both feet simultaneously. In the rigid coalition foot, the heel remains everted or vertical because of the rigid nature of the condition; conversely, in a flexible foot, the heel will invert and reduce because the equinus component is negated, allowing the extrinsic musculature to realign the foot [14]. When supination of the weight-bearing foot generates only a limited external rotation of the tibia, tarsal coalition must be assumed [26]. The ‘step-forward Hubscher maneuver’ was introduced as an effective means of evaluating the flexibility and reducibility of a pes planovalgus deformity by negating the effects of a gastrocnemius or gastrocnemius-soleus equinus. In stance, the hallux is manually dorsiflexed and the leg is externally rotated, which will demonstrate a restored arch in the flexible/reducible foot, via windlass loading of the plantar fascia. Conversely, this maneuver will fail to restore the arch in a rigid foot [8].

### Radiographic imaging

**Figure 1:**
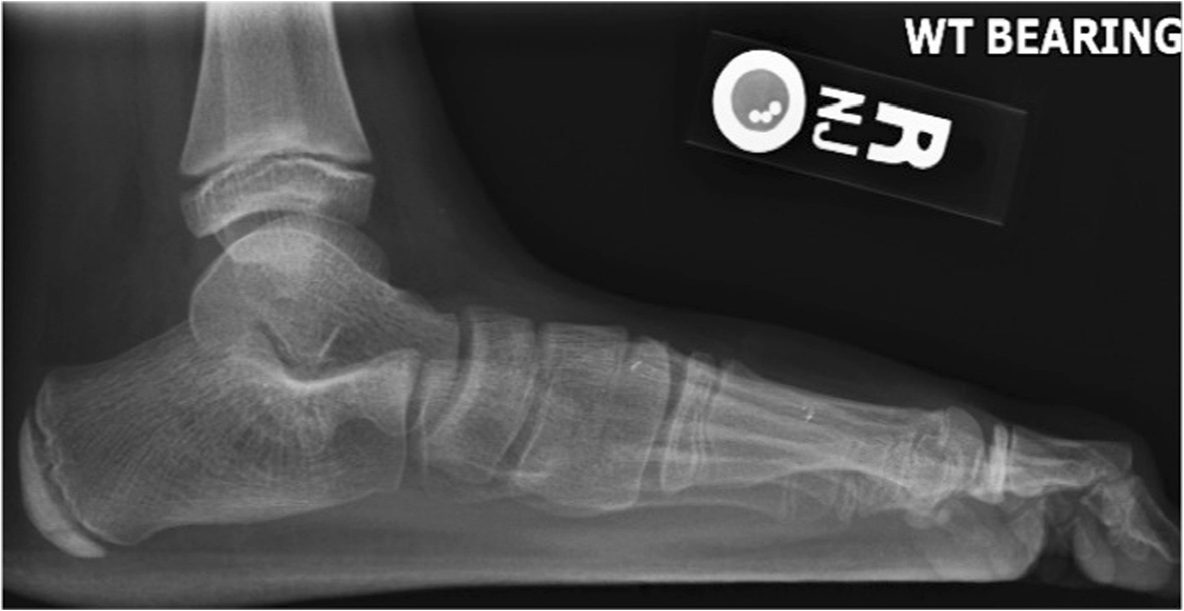
**The lateral X-ray image shows the C sign.**

Bony coalition can be detected on routine radiographs. Talocalcaneal coalitions had an 88% diagnostic specificity radiographically [27]. The C sign has been described as an important radiographic sign of talocalcaneal coalition which is a C-shaped line created by the outline of the talar dome and the inferior margin of the sustentaculum tali [26]. A middle facet coalition in the subtalar joint results in uneven medial forces through the middle facet, rather than the posterior facet, which normally bears the load of these forces. Therefore, it is seen that there is irregularity of the middle facet and hypertrophy of the sustentaculum in the lateral radiograph. The anatomic-pathologic results showed that the ‘C’ sign is a bony bridge between the talar dome and sustentaculum tali, in combination with a prominent inferior border of the sustentaculum tali (Figure 1). Brown et al. have shown that the ‘C’ sign may be more indicative of collapsing flatfoot deformity than coalition [26]. In addition, the fibula may come into contact with the lateral calcaneal wall during weight bearing and develop a pseudoarticulation in the presence of calcaneal valgus [19].

**Figure 2:**
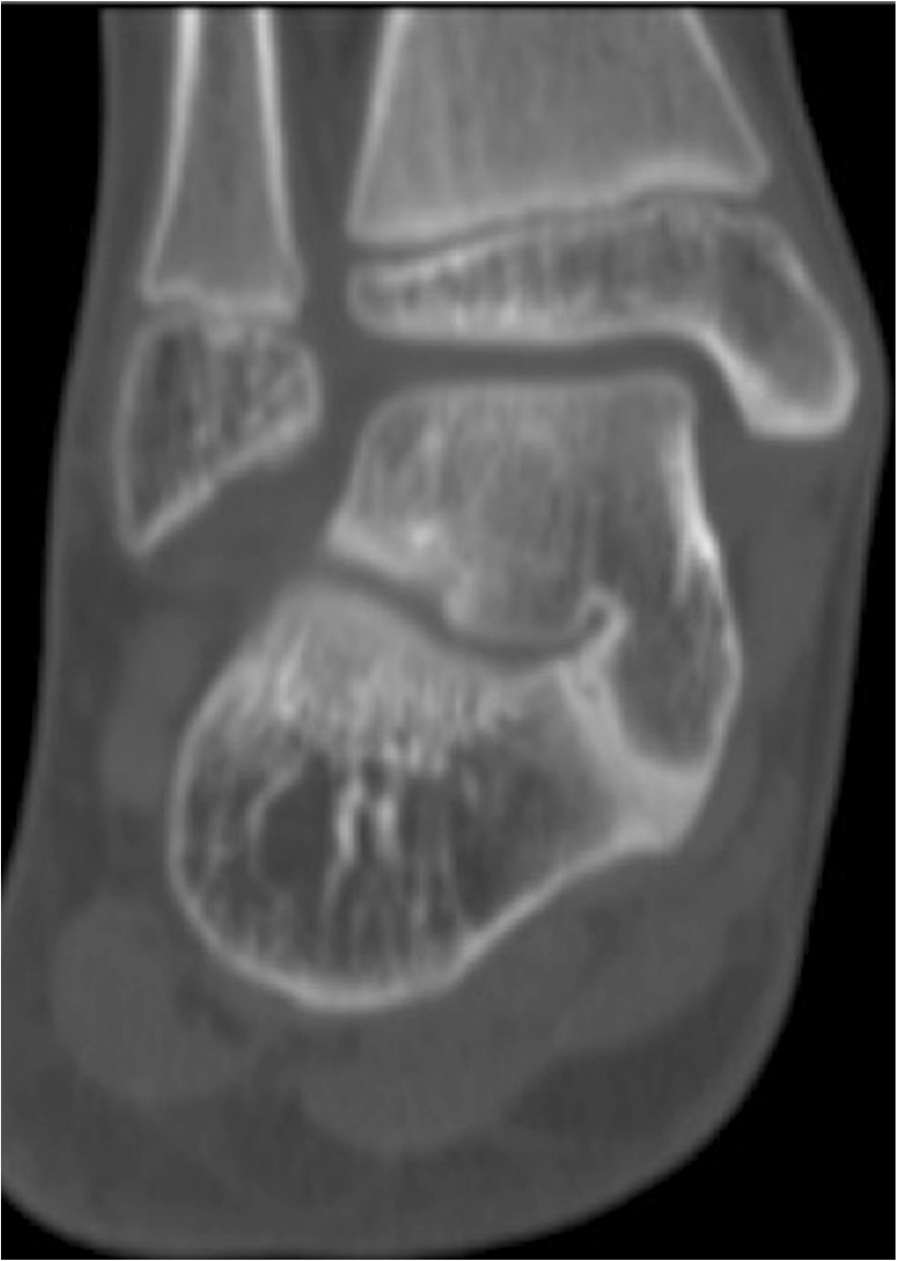
**The image of CT shows a bony talocalcaneal coalition.**

Fibrous or cartilaginous talocalcaneal coalitions may be difficult to visualize on the three standard radiographic views of the foot. CT and MR imaging will help to delineate the diagnosis and guide the surgical treatment plan [9,28]. CT and MR imaging allow differentiation of osseous from nonosseous coalitions and depict the extent of joint involvement as well as secondary degenerative changes [27] (Figure 2). On MR images, bone marrow edema, abnormal articular orientation, and joint space narrowing were frequently identified adjacent to the abnormal joint. However, Wilde showed that there was a significant association between the outcome scores and the area of coalition [29]. Talocalcaneal coalitions with an associated flatfoot deformity may be detected with advanced imaging when the physical exam and standard radiographs are equivocal.

## Treatment options

The goal of conservative treatment for patients with talocalcaneal coalition and flatfoot is to limit subtalar and midtarsal joint range of motion in an effort to reduce pain and muscle spasms. Conservative treatments include arch supports, a short-leg walking cast, immobilization in neutral or a slight varus position, and anti-inflammatory medications [2,7]. Thirty percent of the patients were pain free after 6 weeks of cast treatment in one study [30]. Failure of conservative treatment in symptomatic talocalcaneal coalitions with flatfoot usually mandates a surgical intervention.

Currently, surgical techniques including bar resection with or without interposition in the resection gap, subtalar joint arthrodesis, and also extra-articular arthrodesis in the sinus tarsi have been reported [31,32]. Though, Khoshbin reported that no association was noted between the size of talocalcaneal coalition with respect to outcome scores [33]. It is generally accepted to perform a single resection of coalition for intractably painful small talocalcaneal coalition that is associated with a wide, healthy posterior facet and minimal valgus deformity of the hindfoot. However, most of coalitions in talocalcaneal middle facet are associated with rigid pes planovalgus. Simple resection is not suitable for all talocalcaneal coalition cases due to the variation in anatomy and deformity.

The traditional medial incision to the subtalar joint provides excellent exposure and resection of the coalition [34–37]. However, the traditional open technique had some disadvantages including the risk of incisional neuroma formation, superficial wound infection, delayed wound healing, and prolonged hospitalization for wound management and pain control [38].

Recently, posterior arthroscopic technique for posterior-facet talocalcaneal coalition excision has been developed [38,39]. In the prone position, Bonasia introduced two portals, posterolateral (PL) portal and posteromedial (PM) portal for resection of talocalcaneal coalition [38]. Lee introduced a third portal, which was defined 1 cm proximal and 1 cm posterior to the tip of the lateral malleolus [40]. Beimers introduced another accessory portal, sinus tarsi portal, for introduction of a large diameter blunt trocar [41]. In the lateral position, generally anterior, medial, and posterior portal are used [42,43]. The anterior and middle subtalar portals were both less likely to damage the sural nerve and its branches than the posterior subtalar portal [44]. The arthroscopic procedure provides a better visualization of pathologic changes in the talocalcaneal joint and a complete resection of coalition [45,46].

**Figure 3:**
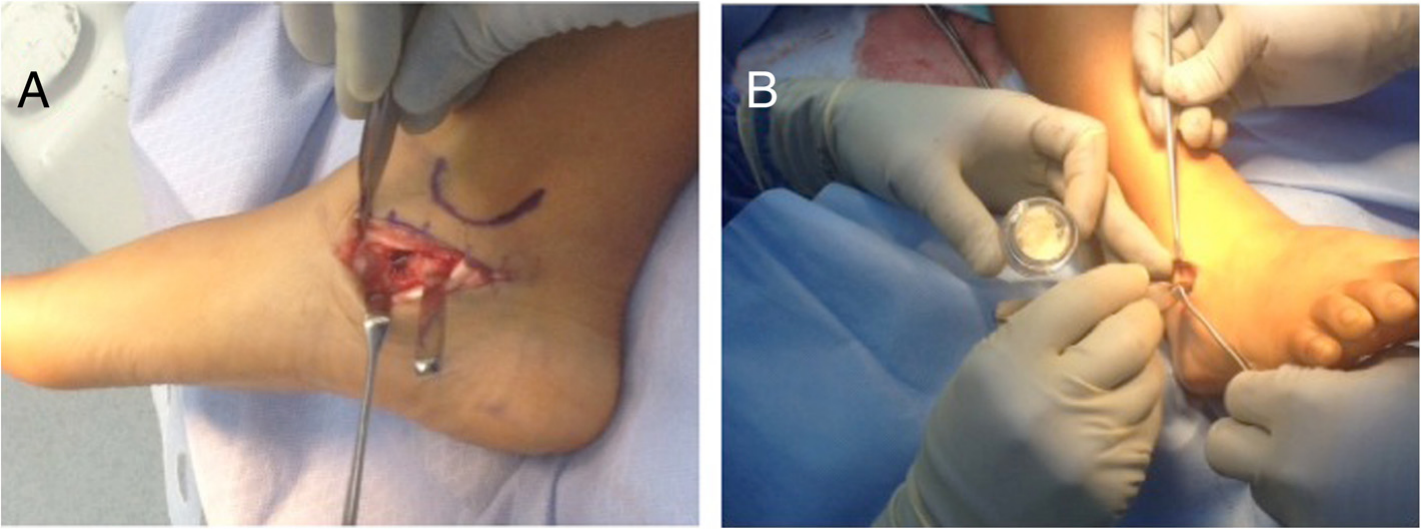
**Incision for the exposure of the subtalar joint and the coalition. (A)** shows the medial incision for the resection of coalition; **(B)** shows the lateral incision for sinus tarsi arthrodesis with Cloward spinal graft.

Senior authors (MAH and KLT) use a medial approach for an isolated coalition resection; and perform a dual lateral and medial approach for resection of the coalition and sinus tarsi arthrodesis, respectively (Figure 3A, 3B). We found no skin or nerve complications with either our isolated or dual incisional approaches. In conclusion, both open surgery and arthroscopy provide good exposure that facilitates predictable results.

The majority of patients with tarsal coalition showed a symptomatic relief and functional improvement in the short term after single resection of coalition [20,30,47]. However, patients with talocalcaneal coalition and flatfoot deformity may present with recurrent pain and worsening planovalgus deformities after a single resection of coalition. Hindfoot valgus and pes planovalgus deformities did not correct well after single resection of talocalcaneal coalitions [33,48]. These are due to the secondary effect of soft tissue contractures (lateral ligaments, peroneal tendons, calf muscles) ‘pulling’ the foot into more valgus [49]. Normal plantar pressures were not recreated during running after resection of tarsal coalition [20,50]. In view of normal subtalar kinematics playing an important role with long-term implications; resection of the coalition combined deformity correction may be more appropriate in the patients, who had a talocalcaneal coalition with flatfoot deformity. In 1987, Olney performed excision of the coalition and an autogenous free fat graft for nine patients with a symptomatic talocalcaneal joint. Evaluation revealed five excellent, three good, one fair, and one poor result after 42 months follow-up postoperatively [51]. Interposition of the split flexor hallucis longus tendon resulted in considerable relief of pain, an improved range of motion of the talocalcaneal joint, and improved function of the foot after resection of talocalcaneal coalition [52,53]. In addition, an interposition with a tensor fascia lata allograft [54] or extensor digitorum brevis [55] or fibrin glue [56] achieved excellent or good results as well.

Single-stage middle facet talocalcaneal coalition resection combined with flatfoot reconstruction achieved a significant improvement in radiography and function of the foot [48]. Giannini reported that arthroereisis by implanting a bioreabsorbable device after resection of the tarsal coalition seems to restore the alignment of the hindfoot and reduce pain effectively for symptomatic flatfoot associated with talocalcaneal coalitions [57]. Arthroereisis functions by a combination of mechanical and proprioceptive effects that allow for growth adjustment of the subtalar joint and with a low complication rate. Though arthroereisis is contraindicated for treating fixed and secondary pes planovalgus [58]; the foot may gain mobility, albeit still limited, once resection of the talocalcaneal coalition has been performed. Gougoulias thought that gastrocnemius, Achilles, and/or peroneal tendon releases should be performed when the foot is in a planovalgus appearance, and also corrective calcaneal osteotomy may be required for young and adolescent patients with a flexible deformity to avoid equinus or further recurrence [49]. Mosca has reported that calcaneal lengthening osteotomy with gastrocnemius or Achilles tendon lengthening effectively corrected deformity and relieved pain in talocalcaneal coalition with planovalgus foot [59].

**Figure 4:**
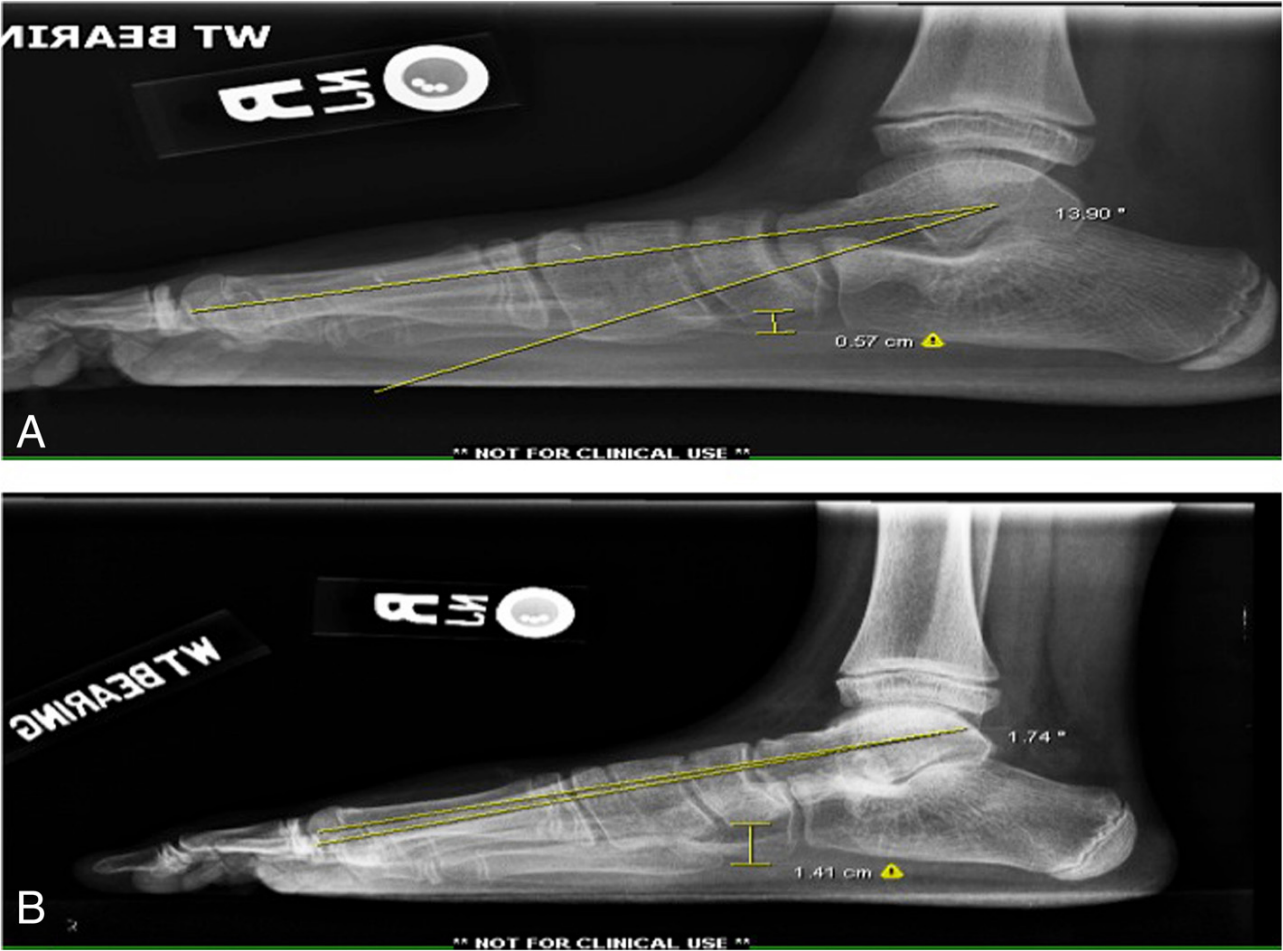
**The weight-bearing lateral X-ray shows that the angle of the first tarsometatarsal and the height of the arch in the foot improved (A and B).**

**Figure 5:**
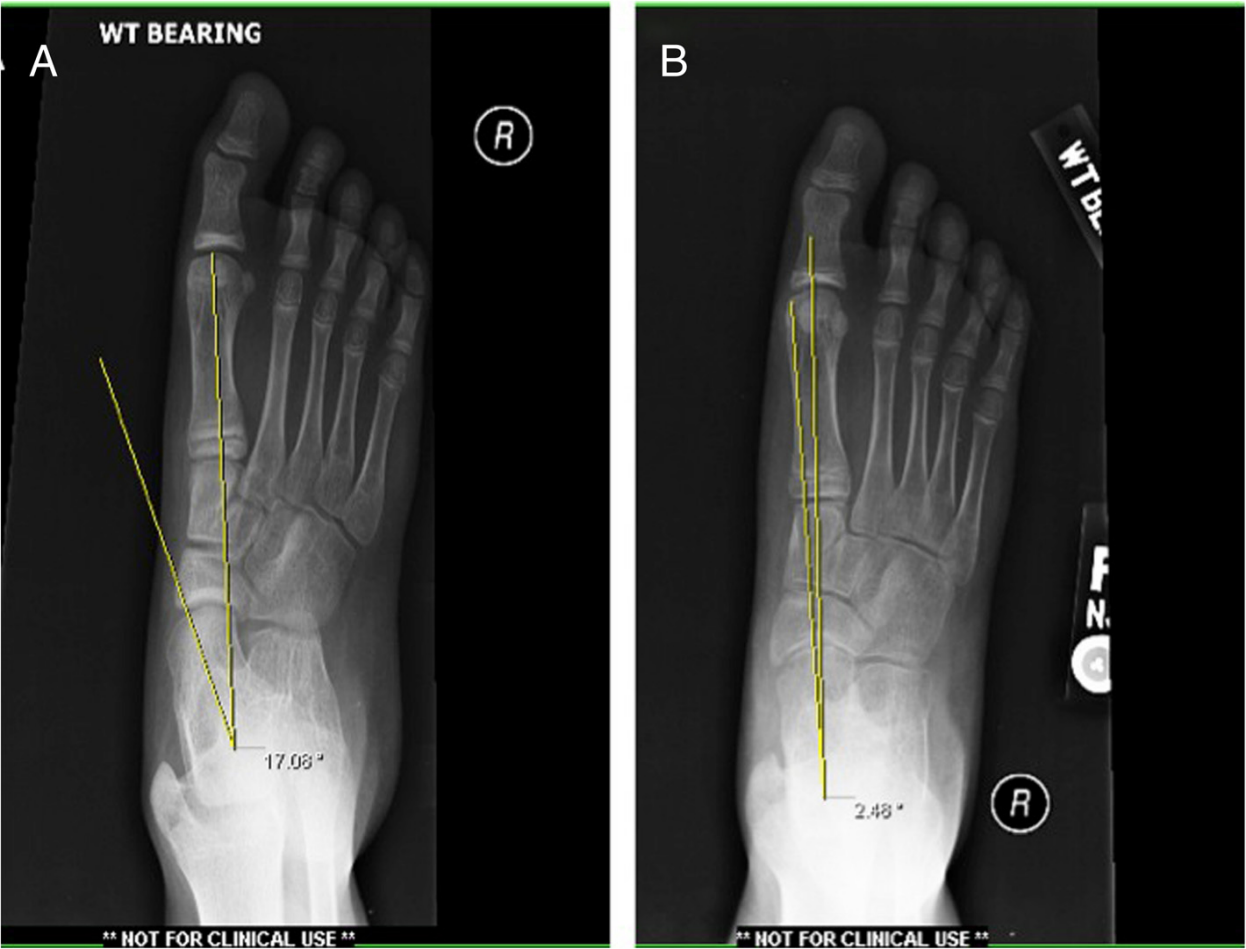
**The weight-bearing anteroposterior X-ray shows that the angle of the first tarsometatarsal improves (A and B).**

According to our experience, middle facet coalitions comprising more than 50% of the middle facet are best served with a subtalar joint arthrodesis. However, caution should be taken with primary arthrodesis of the entire talocalcaneal complex in children under the age of 13 due to possible growth disturbances of the hindfoot complex. An extra-articular arthrodesis is favored to prevent stunting of the normal hindfoot development. The Grice procedure has been originally designed by Grice in the treatment of children with polio [60]. Grice procedure performed extra-articular subtalar arthrodesis, which does not affect further bone growth and was widely used to correct spastic valgus deformity in children decades ago [61]. One of the senior authors (MAH) frequently performs modified extra-articular arthrodesis in osseous juvenile tarsal coalitions with the use of a Cloward spinal graft with much success (Figures 4 and 5). In patients where less than 50% of the middle facet is involved, an attempt at salvage of the subtalar joint is preferred. This most often involves the resection of the coalition with placement of a subtalar joint arthroereisis to realign the hindfoot and preserve motion. Bone-block distraction was used in sinus tarsi arthrodesis. With bone-block distraction under the body of the talus, near-anatomical talar inclination is restored.

Collectively, nonoperative treatment may prove successful in children with talocalcaneal coalition with flatfoot. Resection of the coalition combined with an interpositional graft or arthroereisis or sinus arthrodesis has shown favorable outcomes. There is currently no clear consensus on the operative algorithm for talocalcaneal coalition with flatfoot. However, two operative criteria should be noticed for a long-term outcome: first, do not disturb the growth of the talocalcaneal joint; second, to correct the deformity combining resection of the coalition in a single-stage operation.

## Conclusion

Talocalcaneal coalition with flatfoot is commonly seen in children. Talocalcaneal coalitions become increasing symptomatic as they ossify and restrict subtalar motion. Talocalcaneal coalition combined with a rigid flatfoot is characterized by some unique symptoms upon physical examination. Routine plain-film radiographs may detect talocalcaneal coalition combined with flatfoot effectively; however, advanced imaging such as CT and MRI may prove beneficial to delineate the etiology and guide surgical treatment. Conservative treatment options are useful and should be exhausted prior to entertaining surgical reconstruction. The optimal surgical procedure for talocalcaneal coalition with rigid flatfoot has not been definitively determined. However, combining resection of the coalition with concomitant flatfoot correction in a single-stage operation has shown to be valuable according to the long-term results.
